# Pleuritis associated with immunoglobulin G4-related disease under normal thoracoscopic findings: a case report

**DOI:** 10.1186/s13256-021-02718-4

**Published:** 2021-04-30

**Authors:** Hiroki Shimada, Yuto Kato, Miyuki Okuda, Koji Fukuda, Nobuya Tanaka, Yutaro Okuda, Akihiko Yoshizawa

**Affiliations:** 1 Hirakata Kohsai Hospital, 1-2-1, Fujisakahigashimachi, Hirakata, Osaka 573-0153 Japan; 2 Department of Respiratory Medicine, Nara Medical University Hospital, 840 Shijocho, Kashihara, Nara 634-8522 Japan; 3 Department of Diagnostic Pathology, Kyoto University Hospital, 54 Shougoin-Kawaharacho, Sakyo-ku, Kyoto, 606-8507 Japan

**Keywords:** IgG4-related disease, Medical thoracoscopy, Pleuritis

## Abstract

**Background:**

Immunoglobulin G4 (IgG4)-related disease is a chronic inflammatory disease that was recognized in 2011. Pleuritis associated with IgG4-related disease is rare and can be difficult to diagnose. Although there have been previous reports on pleuritis associated with IgG4-related disease by thoracoscopic findings, this is the first to observe pleuritis with IgG4-related disease from normal pleural thoracoscopic findings.

**Case presentation:**

A 70-year-old Japanese female treated for breast cancer 33 years ago was referred to our hospital complaining of dyspnea on exertion. Chest computed tomography (CT) revealed left pleural effusion that was exudative and predominant with lymphocytes, elevated adenosine deaminase (ADA) and Class III cytology (malignancy suspected). Subsequently, thoracoscopic pleural biopsy was performed for definitive diagnosis. Although pleural macroscopic findings appeared normal, we performed pleural biopsy at random sites. This patient was negative for mycobacterium tuberculosis, and neither granulomas nor malignant cells were found in the collected specimens. An infiltration of inflammatory cells, mainly plasma cells and lymphocytes, was observed. Immunostaining revealed the number of IgG4-positive plasma cells was 102/high power field (HPF), and the percentage of IgG4 positive/immunoglobulin G (IgG)-positive cells was 41.4%. Since IgG4 serum levels were high and IgG4-related submandibular sialadenitis was also observed, a definitive diagnose of pleuritis associated with IgG4-related disease was confirmed.

**Conclusions:**

We diagnosed pleuritis associated with IgG4-related disease by thoracoscopic pleural biopsy samples taken from a visually normal pleura. Although exudative pleural effusion with high ADA and lymphocyte predominance is a characteristic of tuberculous pleuritis, other diseases might be present. Since thoracoscopy can increase the diagnostic yield, pleural biopsy should be considered even if thoracoscopic pleural findings are deemed normal.

## Introduction

Immunoglobulin G4 (IgG4)-related disease is a chronic inflammatory systemic disease that was recognized in 2011 [1]. IgG4-related disease, a multi-organ disease, is known to cause damage to the pancreas, bile duct, lacrimal gland, salivary gland, central nervous system, thyroid, lung, liver, digestive tract, kidney, prostate, retroperitoneum, artery, lymph node, skin, and mammary gland. Among these, lung lesions are particularly difficult to diagnose, requiring computed tomography (CT)-guided biopsy or thoracoscopy for diagnosis. On the other hand, reports of pleuritis associated with IgG4-related diseases are rare, and only around 20 cases have thus far been reported. Pleural findings such as pleural plaque, fibrous deposits, pleural thickening, and nodules are recognized as IgG4-related disease, but currently, there are no reports on IgG4-related disease from normal plural findings assessed by thoracoscopy.

We report a case of pleuritis associated with IgG4-related disease confirmed by randomly selected pleural biopsy sites after normal thoracoscopic findings.

## Case presentation

A 70-year-old Japanese female complaining of dyspnea on exertion was admitted to our hospital after chest X-ray showed left pleural effusion. Her medical history included hypertension, dyslipidemia, paroxysmal atrial fibrillation, cerebral infarction, and left breast cancer. Her hypertension, dyslipidemia, and paroxysmal atrial fibrillation were well managed by medication, and she underwent total left mastectomy and remnant gauze removal surgery 33 and 13 years ago, respectively. She was a nonsmoker with no history of autoimmune diseases, multiple myeloma, tuberculosis, or exposure to asbestos. Her family history revealed that her father had liver cirrhosis, and her mother had been diagnosed with lung cancer. There was no family history of autoimmune disease or tuberculosis.

On admission, she was lucid, with heart rate of 74 beats/minute, blood pressure of 138/68 mmHg, peripheral oxygen saturation of 96%, and body temperature of 35.8 °C. Heart sounds were normal but with decreased breath sounds in the left lung field. No spontaneous pain or tenderness was present in the chest. Her abdomen was flat and soft, with no abdominal tenderness or bowel sounds. No pitting edema was observed in either her forearms or legs; however, bilateral submandibular gland enlargement was observed. She had no motor or sensory deficits in her extremities and no arthralgia or morning stiffness associated with rheumatoid arthritis.

### Laboratory findings

**Fig. 1.**
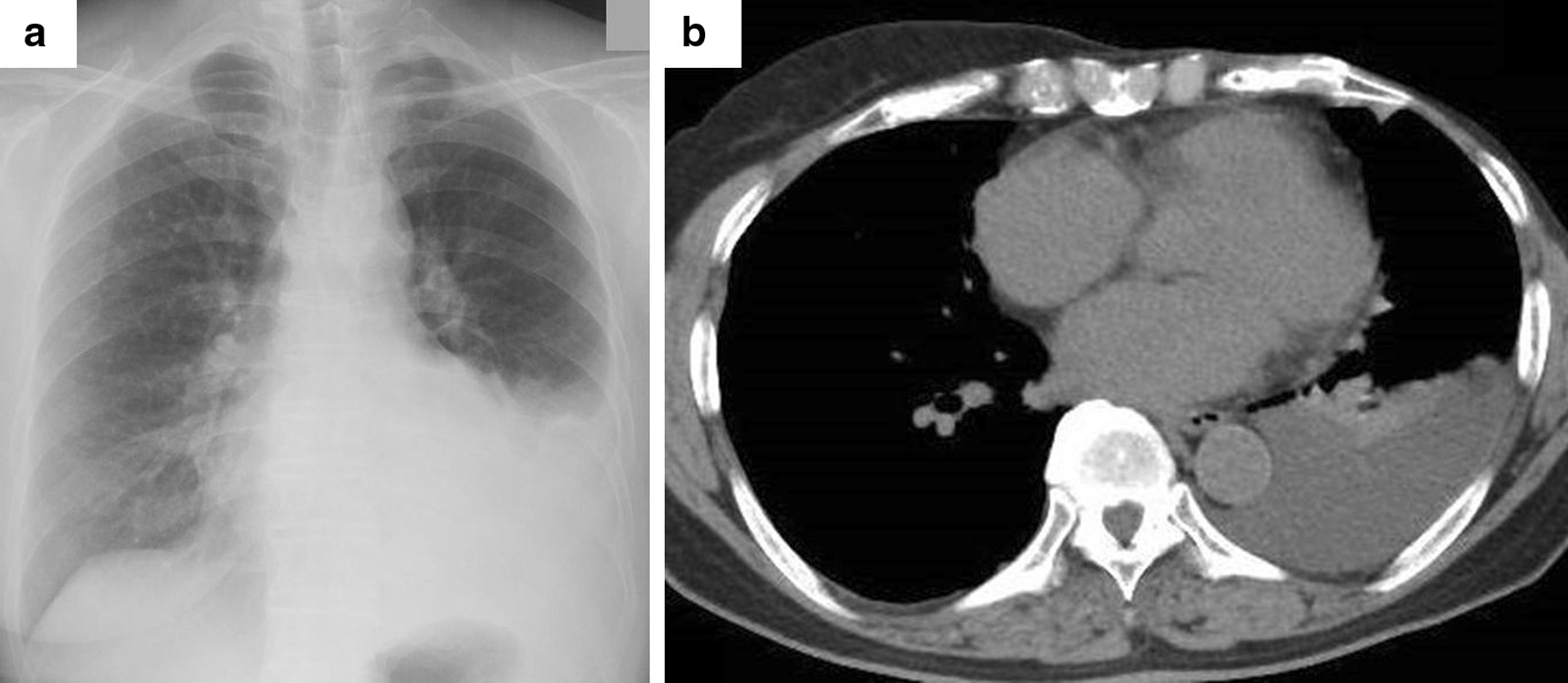
Radiological findings. Chest X-ray at admission showed pleural effusion (**a**). Chest CT scan showed no thickening, calcification, or nodules in the left pleura. No tumour nodules were suspected in the lungs (**b**)

Inflammatory findings such as C reactive protein (CRP) and tumour makers were within the normal range (normal CRP, < 0.3 mg/dL). Autoantibodies were serologically negative. Serum immunoglobulin G (IgG) was 2001 mg/dL including 637 mg/dL for IgG4 (normal IgG 870–1700 mg/dL, normal IgG4 4–108 mg/dL). Chest X-ray and CT showed left pleural effusion without abnormal lung shadow and no pleural thickening (Fig. 1a, b). We performed pleural puncture and drained 1200 mL of pleural fluid. Pleural puncture revealed lymphocytes were dominant and adenosine deaminase (ADA) was high, but *Mycobacterium tuberculosis* was not detected (normal ADA 8.6–20.5 U/L). Elevated IgG and IgG4 levels were observed in the pleural effusion (IgG 1756 mg/dL; IgG4 684 mg/dL). Class III cells were identified from the pleural effusion, suggesting the possibility of breast cancer dissemination. Mycobacterium cultures for sputum, pleural fluid, and pleural biopsy samples were negative.

### Clinical progress

**Fig. 2.**
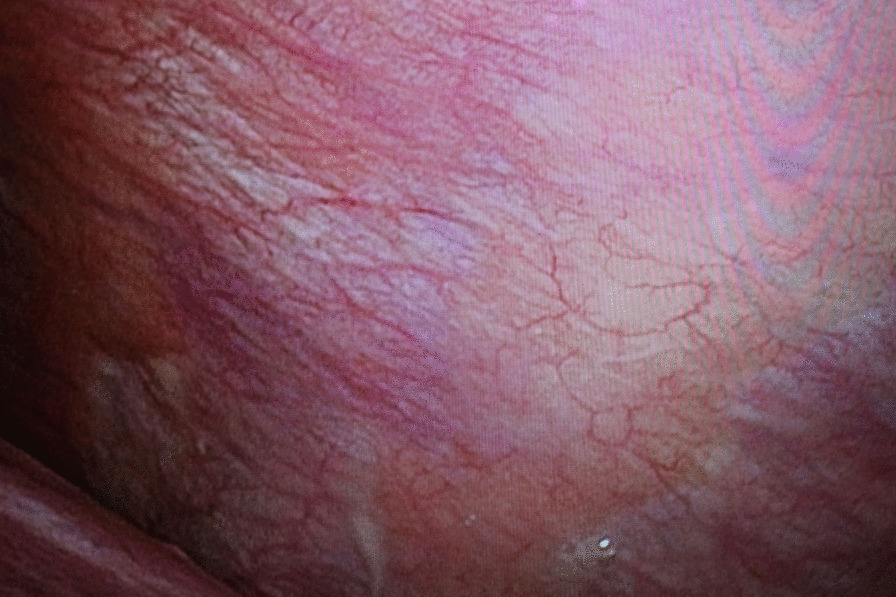
Thoracoscopic findings of left pleura. Thoracoscopically, there were no obvious mass lesions, pleural thickening, nodules, or pleural-pulmonary adhesions observed for the left pleura

The results of pleural puncture suggested tuberculous pleuritis, pleuritis due to autoimmune disease, or carcinomatous pleuritis, but a definitive diagnosis could not be made. Thus, we performed thoracoscopic pleural biopsy. Under thoracoscopy, no obvious lesions, pleural thickening, nodules, or pleural-pulmonary adhesions were found for the left pleura. Although the pleura appeared normal, we randomly collected seven specimens at three locations (Fig. 2).

**Fig. 3.**
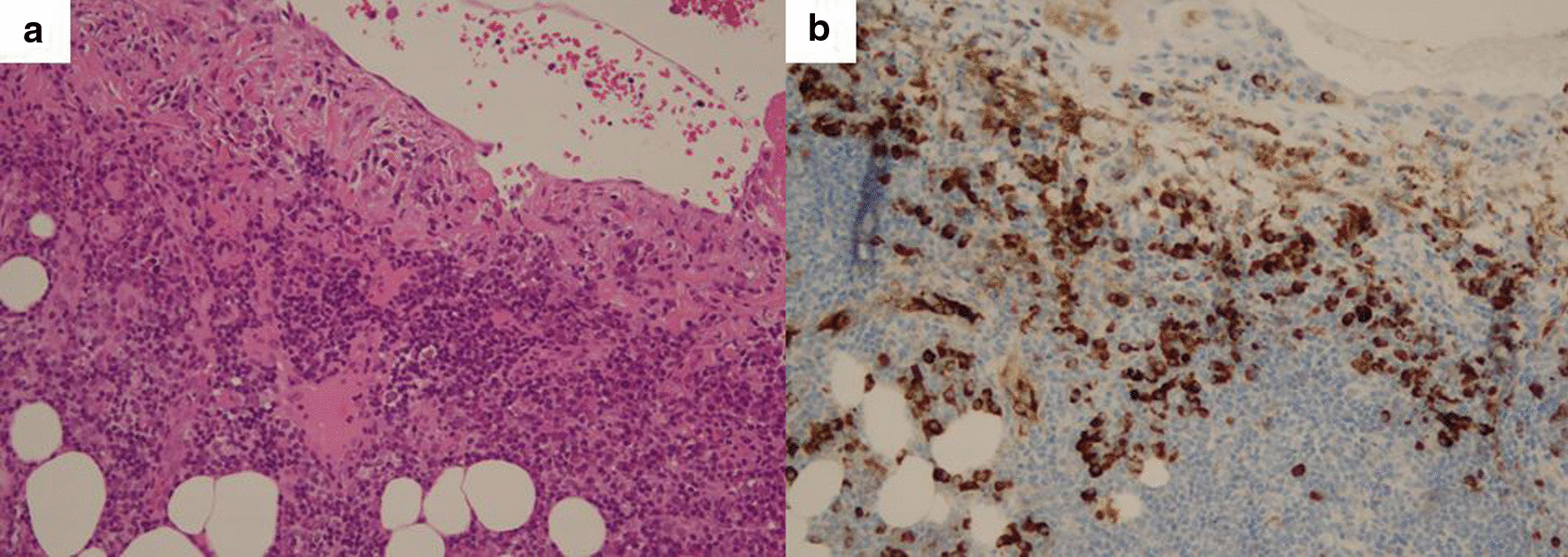
Pathological findings of the left pleura. Biopsy specimens stained with hematoxylin and eosin stain (**a**) and IgG4 immunostaining (**b**). Plasmacytoid, lymphocyte-dominated inflammatory cell infiltration was present. The formation of reactive lymphatic follicles was also found

Pathological examination revealed membranous tissue with superficial proliferation in the reactive mesothelium. We confirmed plasmacytoid, lymphocyte-dominated inflammatory cell infiltration. The formation of reactive lymphatic follicles was also present. Polymerase chain reaction for mycobacterium tuberculosis (TB-PCR) was negative, and no granulomas were found in the pleural specimens. There were no malignant epithelial lesions and immunostaining showed no deviation in IgG-k or IgG-λ chains. Epstein-Barr virus-encoded small Rino Nucleic Acid (RNA) and Congo red stain were also negative. IgG4-positive cells were 102/high power field (HPF), and the percentage of IgG4 positive/IgG positive cells was 41.4%, suggesting pleuritis associated IgG4-related disease (Fig. 3a, b).

We then performed 2-deoxy-2-(18F]-fluoro-d-glucose (FDG) positron emission tomography (PET)-CT for systemic retrieval analysis. FDG PET-CT showed FDG accumulation within the bilateral submandibular glands of the left pleura. Subsequently, under ultrasound guidance, we collected biopsy samples from the right side of the submandibular gland. An infiltration of plasmacytes and lymphocytes around the adeno-atrial tissue was present; however, no malignant cells or granulomas were noted. Immunostaining confirmed IgG4-positive cells were 96/HPF and the percentage of IgG4 positive/IgG positive cells was 55.2%, confirming pleuritis-associated IgG4-related disease.

We have started treatment with prednisolone 30 mg/day because of increased pleural effusion after discharge from the hospital. The patient was followed for 10 months. The dose was reduced every month from 30 to 20 mg/day to 15 mg/day to 12.5 mg/day to 10 mg/day. Eventually, the dose was reduced to 7.5 mg/day 6 months after the start of treatment, and now the dose was continued at 7.5 mg/day. Since the prednisolone dose was reduced, the patient has progressed without relapse of pleuritis and submandibular gland swelling. Although some side effects of prednisolone such as lipid abnormalities and worsening of diabetes were observed, the treatment was successful without major side effects.

## Discussion

In this case, a biopsy from a grossly normal pleura led to the diagnosis of pleuritis associated with IgG4-related disease, and remission was achieved with steroid therapy. Although papers on IgG4-related diseases have reported abnormal findings in the pleura [2–4], we were able to confirm a definitive diagnosis of pleuritis associated with IgG4-related disease by performing random biopsies of the pleura using thoracoscope. IgG4-related disease is a chronic inflammatory condition characterized by mass or hypertrophic lesion infiltration with lymphocytes and IgG4-secreting plasma cells with various degrees of fibrosis. The clinical diagnostic criteria for IgG4-related disease states: (1) clinical examination showing characteristic diffuse/localized swelling or masses in single/multiple organs; (2) hematological examination of elevated serum IgG4 concentrations (> 135 mg/dL); (3) histopathologic examination showing (a) marked lymphocyte and plasmacyte infiltration and fibrosis and (b) infiltration of IgG4-positive plasma cells: ratio of IgG4-positive/IgG positive cells > 40% and > 10 IgG4-positive plasma cells/HPF [5]. This present case satisfied all of the aforementioned criteria.

Additionally, the diagnostic criteria for IgG4-related respiratory disease were proposed as follows: (1) abnormal shadow on chest CT; (2) serum IgG4 level of > 135 mg/dL; (3) histopathologic features fulfilling the comprehensive diagnostic criteria; (4) presence of extrathoracic lesions [6]. IgG4-related diseases can cause interstitial pneumonia, inflammatory nodules, and airway inflammation, but pleuritis is uncommon.

Pleural effusion for heart and renal failures is often due to systemic diseases associated with leakage or bilateral pleural effusion. Pleural effusion due to IgG4-related disease is expected to be exudative pleural effusion due to inflammation and can take the form of either unilateral or bilateral pleural effusion patterns. Pleural effusion in pleuritis associated with IgG4-related disease has been reported to be lymphocyte-dominated exudative pleural effusion with high ADA [7]. To date, lymphocyte-dominated exudative pleural effusion with a high level of ADA has been considered an indication of tuberculous pleuritis. A report on ADA [8] showed a sensitivity of 100% and a specificity of 93.9% for tuberculous pleuritis if the cut-off value of ADA was set for 40.3 U/L, after which, many studies have shown the diagnostic accuracy of tuberculous pleuritis to be high [9, 10]. To diagnose tuberculous pleuritis, it is important to confirm by mycobacterium culture and TB-PCR. Other diagnostic tools are cell fractionation of the pleural fluid, pleural fluid ADA activity, QuantiFERON TB-2G (QFT-2G; Cellestis Ltd., Carnegie, Victoria, Australia), and purified protein derivative (PPD) reaction.

However, Miyoshi et al. reported that only 1 of 32 cases of tuberculous pleuritis had a positive pleural fluid culture, and it is thought that the diagnosis based on pleural fluid examination alone is extremely difficult. Pleural biopsy, which was performed in this case, is said to be useful for diagnosis and has superior sensitivity and specificity compared to pleural fluid examination [11]. In tuberculous pleuritis, pleural gross findings often show nodular lesions and pleural thickening, and pathology proves butyric granulomas. Tuberculous pleuritis was considered negative since this case showed elevated ADA but no evidence of mycobacterium tuberculosis on pleural fluid culture or pleural biopsy sample, and the gross findings of pleura were normal. Pathology did not reveal butyric granulomas, and her TB-PCR was negative.

On the other hand, exudative pleural effusion with high ADA and lymphocyte-dominated pleural effusion is not a finding specific to tuberculous pleuritis since it is also found in primary effusion lymphoma, leukemia, pyothorax, rheumatic disease, and pleural effusion associated with malignant pleural mesothelioma. It has been reported that thoracoscopic pleural biopsy can provide a definitive diagnosis 90% of the time [12, 13]. Therefore, for our case, a diagnosis of pleuritis associated IgG4-related disease obtained from pleural biopsy is considered quite accurate.

Thus far, three patterns of thoracoscopic findings have been described for IgG4-associated pleuritis: (1) milky pleural plaques suggestive of hyalinized collagen fiber deposits; (2) diffuse inflammatory thickening of the pleura; (3) nodules on the parietal pleura [2]. In each of these cases, pleural biopsies showed an infiltration of IgG4-positive cells. However, in our case, the thoracoscopic findings were normal, but random biopsies led to a definitive diagnosis. Pleural biopsy is considered useful for diagnosis and should be performed even when thoracoscopic findings of the pleura appear normal.

## Conclusions

Although thoracoscopic findings of the pleura appeared normal in this case, we performed random pleural biopsies to confirm a diagnosis of pleuritis associated with IgG4-related disease. We suggest that thoracoscopic pleural biopsy should be considered even when pleural findings on thoracoscopy appear normal.

## Data Availability

The authors declare that the data supporting the findings of this case report are available within the article and its additional information files.

## Abbreviations

IgG4: Immunoglobulin G4
IgG: Immunoglobulin G
CT: Computed tomography
ADA: Adenosine deaminase
FDG: 2-Deoxy-2-[18F]-fluoro-d-glucose
PET: Positron emission tomography
HPF: High power field
TB-PCR: Polymerase chain reaction for *Mycobacterium tuberculosis*
QFT-2G: QuantiFERON TB-2G
PPD: Purified protein derivative
